# It’s the Little Things (in Viral RNA)

**DOI:** 10.1128/mBio.02131-20

**Published:** 2020-09-15

**Authors:** Jiří František Potužník, Hana Cahová

**Affiliations:** a Institute of Organic Chemistry and Biochemistry of the Czech Academy of Sciences, Prague, Czech Republic; Albert Einstein College of Medicine; University of Texas Health Science Center at Houston

**Keywords:** RNA modification, RNA modification detection, RNA virus, retroviruses, viral RNA

## Abstract

Chemical modifications of viral RNA are an integral part of the viral life cycle and are present in most classes of viruses. To date, more than 170 RNA modifications have been discovered in all types of cellular RNA. Only a few, however, have been found in viral RNA, and the function of most of these has yet to be elucidated. Those few we have discovered and whose functions we understand have a varied effect on each virus. They facilitate RNA export from the nucleus, aid in viral protein synthesis, recruit host enzymes, and even interact with the host immune machinery. The most common methods for their study are mass spectrometry and antibody assays linked to next-generation sequencing. However, given that the actual amount of modified RNA can be very small, it is important to pair meticulous scientific methodology with the appropriate detection methods and to interpret the results with a grain of salt. Once discovered, RNA modifications enhance our understanding of viruses and present a potential target in combating them. This review provides a summary of the currently known chemical modifications of viral RNA, the effects they have on viral machinery, and the methods used to detect them.

## Abstract (precis)

Chemical modifications of viral RNA are an integral part of the viral life cycle and are present in most classes of viruses. To date, more than 170 RNA modifications have been discovered in all types of cellular RNA. Only a few, however, have been found in viral RNA, and the function of most of these has yet to be elucidated. Those few we have discovered and whose functions we understand have a varied effect on each virus. They facilitate RNA export from the nucleus, aid in viral protein synthesis, recruit host enzymes, and even interact with the host immune machinery.

## INTRODUCTION

Viruses are a phylogenetically diverse group of obligate intracellular parasites and it is estimated there are approximately 10^31^ of them in the world today (1). The Baltimore system divides viruses into seven (originally six) main categories based on their genome form: (i) double-stranded DNA (dsDNA); (ii) single-stranded DNA (ssDNA); (iii) double-stranded RNA (dsRNA); (iv) positive single-stranded RNA (+ssRNA); (v) negative single-stranded RNA (−ssRNA); (vi) positive single-stranded RNA retroviruses (ssRNA-RT); and (vii) double-stranded DNA retroviruses (dsDNA-RT) (2, 3). In successful completion of their life cycles, however, these viruses have several things in common. One of them is the creation of viral RNA, whether it be genomic RNA, mRNA, or only an intermediate RNA. As viruses do not have their own translational machinery, they must hijack a host cell apparatus in order to replicate, and they have developed various strategies for this purpose (4–6). Viruses also need to evade host immunity, facilitate RNA export from the nucleus, and improve their RNA stability, translational efficiency, packaging, etc. while avoiding cellular processing of viral RNA. One of the ways they achieve all this is through the use of RNA modifications (7, 8).

Chemical modifications of RNA have been known for over 50 years (9, 10). They affect a wide range of processes, from RNA stability to translational efficiency (11, 12). To date, more than 170 RNA modifications have been identified (13), and our understanding of their functions has improved greatly and been the topic of numerous reviews (14–17). Most of the known chemical modifications are present in rRNA and tRNA. Moreover, the discovery that modifications of mRNA are dynamic and reversible (18) led to the establishment of the new field of epitranscriptomics. Unfortunately, the minuscule amount of regulatory RNA and mRNA, in which these modifications potentially affect the function of the entire RNA molecule, represents a limitation that is difficult to overcome even with the techniques available today (19). The actual number of modifications per mRNA molecule is also rather small. The most abundant modification in mRNA is N^6^-methyladenosine (m^6^A), yet there is only about 1 m^6^A per 1,000 nucleotides (20). Because viral RNA may be more abundant than a given type of cellular mRNA, the difficulties of searching for new RNA modifications in low-abundance RNA species and of understanding their role might be overcome by employing viruses as model systems. Moreover, their simple intrinsic organization may help us understand the role of RNA modifications in virus-derived mRNAs.

The presence of RNA modifications in viral genomic RNA and viral mRNA has a diverse impact on viral machinery. Modifications on the Watson-Crick face, such as the methylation at position 1 of adenosine (m^1^A) or inosine, change the pairing properties, such that the original base is then read differently by a reverse transcriptase, an RNA-dependent RNA polymerase, or the translational apparatus. The presence of large RNA modifications, e.g., 2-methylthio-N6-threonylcarbamoyladenosine in tRNA, can even stop these processes (21, 22). Several reviews have been written on viral epitranscriptomics, but they tend to focus only on N^6^-methyladenosine (23–35). This review presents a summary of the current findings on viral RNA modifications in general and their effect on the viral life cycle, along with the detection methods used for their discovery. We discuss the effects that chemical modifications in viral genomic RNA and mRNA have on viral infection and attempt to summarize the majority of known methods developed for the detection and identification of RNA modifications, together with the pitfalls that accompany some of the methods.

Given the recent outbreak of the severe acute respiratory syndrome coronavirus 2 (SARS-CoV-2), it is clear that viruses pose a major threat and there is still much to discover about their life cycles and molecular mechanisms. Understanding viral RNA modifications and learning to exploit them may lead to the creation of attenuated vaccines or specifically targeted drugs that could give us an edge in dealing with the next viral pandemic when it strikes.

## VIRAL RNA MODIFICATIONS

### N^6^-methyladenosine.

One of the most abundant chemical modifications of eukaryotic mRNA is N^6^-methyladenosine (m^6^A) (36), which has been shown to be present across virtually all domains of life (37–40). The N^6^-methylation of adenosine is a very dynamic modification. It is added to the RNA by a methyltransferase complex comprising two catalytic subunits (METTL3 and METTL14), a novel protein (KIAA1429), a splicing factor (Wilms’ tumor associated protein [WTAP]), and two other as-yet-unidentified subunits (41) The modification can then be removed by two demethylases—the fat mass and obesity-associated protein (FTO) and AlkBH5 (42)—in a process that regulates RNA metabolism, stability, localization, and protein interactions, as well as transport and splicing (43–45). The methylation is preferentially located at translational start sites, stop codons, and the 3′ UTR (20). It has also been identified as a key component in cancer development and metastasis, as lower levels of m^6^A RNA keep the cell in a pluripotent state, and higher levels drive cellular differentiation (46).

**FIG 1:**
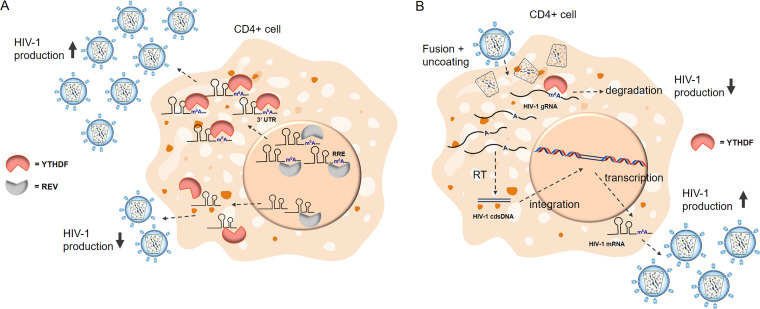
Various mechanisms by which m^6^A in viral RNA influences production of HIV-1. (A) In the nucleus, m^6^A present in the RRE of HIV-1 enhances viral mRNA export (51). In the cytoplasm, m^6^A in the 3′ UTR recruits YTHDF proteins and increases HIV-1 mRNA abundance while improving viral protein translation (49). (B) The m^6^A in HIV-1 genomic RNA leads to RNA degradation, a decrease in reverse transcription, and an overall decrease in infectivity. The m^6^A in the HIV-1 mRNA transcripts, however, results in an increase in viral gene expression (52).

N^6^-methyladenosine is recognized by cytoplasmic readers, the YTHDF proteins (YTHDF1 to YTHDF3). In general, these proteins bind to the modified RNA through their C-terminal YTH domain. YTHDF1 facilitates the translation of modified mRNA and YTHDF2 localizes it to RNA decay sites, while YTHDF3 has a synergistic effect on both (43, 47, 48). In the case of HIV, several laboratories have published results showing different effects of m^6^A on the viral life cycle, suggesting that the role of m^6^A is very complex. The highly conserved YTH carboxy-terminal domain directly binds the m^6^A in the 3′ untranslated region of the HIV mRNA. The overexpression of these proteins greatly enhances viral expression, and their knockdown significantly reduces it (49). The mechanism remains unclear, but YTHDF2 can localize cellular mRNA to RNA decay sites and thus enhance the efficiency of viral mRNA translation (Fig. 1A) (44). Another study, however, showed that YTHDF proteins bound to m^6^A-modified HIV-1 RNA and inhibited genomic RNA and early HIV transcripts, but facilitated viral gene expression in m^6^A-modified late viral transcripts (50). Yet another study showed that m^6^A decreases viral protein expression and viral release (Fig. 1B). On the other hand, based on experiments demonstrating a significant decrease in viral replication upon METTL3 and METTL14 inhibition, along with an increase in viral replication upon AlkBH5 depletion, it seems that m^6^A plays an important role in the regulation of the HIV life cycle (25). This effect may be caused by the HIV-1 Rev protein preferentially binding to a Rev response element (RRE) containing the m^6^A modification and promoting nuclear export of the viral mRNA to the cytosol (Fig. 1A) (51). The effect of m^6^A in the RRE is still under debate, as some studies have shown that m^6^A has a minimal impact on the structure and stability of the RRE. Though the impact of methylation seems marginal, recent small molecule microarray screens have revealed that the change is sufficient for selective recognition by Rev (49, 51–54). The differences in results from studies of m^6^A in HIV can be attributed to several factors, such as the cell type used, the phase of the viral life cycle, the method used to detect m^6^A, etc. These discrepancies are thoroughly discussed in a review focusing specifically on m^6^A (25). It is also important to note that the viral infection can change the abundance of m^6^A in cellular RNA (51). For example, the binding of the CD4 receptor to the HIV-1 envelope glycoprotein GP120 increased the amount of cellular m^6^A by several fold, though the mechanism remains unclear (55).

In comparison to HIV-1 infection, the role of m^6^A during a flaviviral infection is more unambiguous. Based on current studies, m^6^A has an inhibitory effect on flaviviruses such as the hepatitis C virus (HCV) and the Zika virus (ZIKV). An increase in the amount of m^6^A in the viral RNA hinders viral replication and, consistently with this effect, a lowering of m^6^A leads to an increase in viral production (56). In HCV, the E1 gene region showed an ability to bind YTHDF proteins. In infected cells, these proteins subsequently relocalize to the lipid droplets in which viral particle assembly takes place and where they inhibit the packaging of the virus. When YTHDF is overexpressed, it binds to the RNA and hinders viral production. When m^6^A is absent, viral production accelerates due to an increase in HCV core protein binding to the E1 site (57). The YTHDF proteins had a similar effect on the Zika virus. Their knockdown through small interfering RNA (siRNA) led to an increase in ZIKV replication, while their overexpression inhibited the virus, pointing to a conserved mechanism among flaviviruses (58). It is important to note that when a virus infects a cell, it also affects its immune response by modifying the amount of m^6^A present in cellular RNA (34).

### m^5^C.

5-Methylcytosine was first discovered as a chemical modification of DNA more than 70 years ago (59). Its presence in RNA was detected in the late 1970s. Then, ^3^H labeling was used to confirm the presence of m^5^C in the mRNA of hamster cells infected with Sindbis virus (60). More specifically, the viral 26S mRNA coding for viral structural proteins is substantially modified by m^5^C. The 42S mRNA also possesses several m^5^C sites, but significantly fewer than 26S mRNA (61).

In viral RNA, m^5^C has been shown to affect the host innate immune response, binding to the pattern recognition receptor RIG-1 but failing to induce the necessary conformational change that would cause the antiviral signaling cascade (62). The ability of m^5^C to modify viral RNA properties has led some to believe that this modification could facilitate the transfer of viroidal RNA into cellular nuclei or chloroplasts. The presence of m^5^C in viroids, however, was ruled out through bisulfite sequencing (63).

The m^5^C modification has also recently been linked to enhanced retroviral gene expression. In general, the methyltransferase NSUN2 is responsible for the methylation of cytosine in tRNA and mRNA (64), and can also add m^5^C to retroviral transcripts and thus affect their life cycle (65). The genomic RNA of the murine leukemia virus contains as many as 40 m^5^C sites, and their removal inhibits viral replication. Specifically, downregulation of the m^5^C writer NSUN2 through RNA interference (RNAi) caused an overall decrease in Gag protein expression, proving that the presence of m^5^C positively regulates viral replication (66). It has recently been reported that m^5^C is also present in the genomic RNA of SARS-CoV-2 genomic RNA (67). Although the effect of m^5^C on the viral life cycle is clearly visible, more research is necessary to elucidate the mechanisms by which it acts.

### Inosine.

Inosine (I) is an essential modification created through the deamination of adenosine in a process called RNA editing (68). This is done by specific deaminases termed ADAT for tRNA and ADAR for noncoding RNA and mRNA (69). Inosine has been detected in several types of viral RNA, including dsRNA viral transcripts of human herpesvirus 8, negative-sense ssRNA viruses such as human orthopneumovirus, and even virusoids like hepatitis delta virus (HDV) (70–72). The ADARs and the modification itself have been shown to affect the viral life cycle with several mechanisms, either directly by means of the interaction of the modification, or through the inhibition of an immune response against the virus (73, 74). There is a specific isoform of ADAR1, known as p150, which is generated through an interferon (IFN)-inducible alternative promoter, meaning p150 is part of a direct antiviral response (75). ADAR1 has also been shown to positively regulate viral replication by binding and inhibiting the protein kinase R (PKR), which acts as an inhibitor of translation by phosphorylating eukaryotic initiation factor 2 (eIF2α). Phosphorylation of eIF2α stops the cellular mRNA translation and thus prevents the viral mRNA from being translated as well (76). HIV-1 is another example of a virus that actively uses ADAR-1; in fact, ADAR-1 can bind to the HIV-1 p55 Gag protein and is readily incorporated into the virion, pointing to an even more important role of this enzyme and, potentially, inosine in the viral life cycle (77).

**FIG 2:**
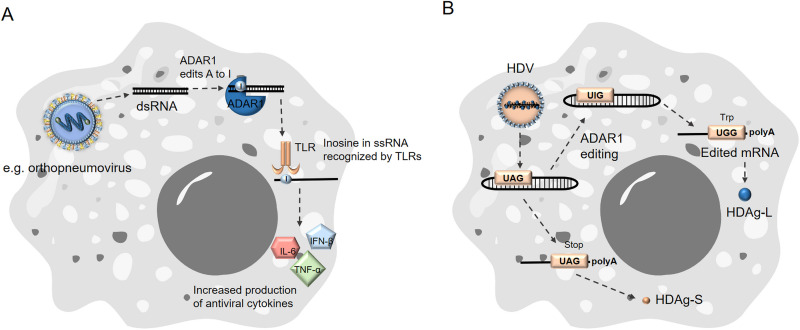
Effect of A to I editing on viral RNA. (A) Orthopneumoviridal negative-sense ssRNA is transcribed into dsRNA by an RNA-dependent RNA polymerase. The dsRNA is then edited by ADAR1 and recognized by Toll-like receptors, which triggers production of antiviral cytokines (e.g., IL-6, IFN-β, and TNF-α). (B) Editing of HDV circular ssRNA by ADAR1 leads to the production of two distinct mRNAs from the same ORF, the unedited HDAg-S and the edited HDAg-L.

In the viral RNA of the human orthopneumovirus (also called the respiratory syncytial virus [RSV]), inosine acts as an innate immune recognition element. An *in vitro*-prepared RNA containing this modification also elicits an immune response. The ssRNA with this modification induces a stronger inflammatory cytokine response (Fig. 2A). It facilitates the release of interferon β (IFN-β), tumor necrosis factor α (TNF-α), and interleukin 6 (IL-6) through binding with a scavenger receptor class-A molecule. This receptor then activates the mitogen-activated protein kinase (MAPK) pathways through toll-like receptor 3 (TLR3). The authors have also demonstrated that inosine-RNA decreases replication of the respiratory syncytial virus (RSV) in epithelial cells *in vitro* (72). It has been suggested that the changes in RNA secondary structures associated with inosine are detected through TLR7 and TLR8. These changes also lead to an increase in TNF-α production, which would mean that inosine serves as a molecular pattern to be recognized in the phagocytosed RNA, as these receptors are mainly expressed by antigen-presenting dendritic cells (78).

Inosine has been shown to play a major role in the life cycle of hepatitis delta virus (HDV). HDV is a negative-strand RNA virusoid that exists in the form of a satellite associated with hepatitis B virus (HBV) (79). HDV produces its two proteins, called the small delta antigen (HDAg-S) and the large delta antigen (HDAg-L), from the same open reading frame (ORF) (80). HDV uses host ADARs, which edit an A to I in the HDAg-S amber codon in the antigenomic RNA. A UIG codon is thus transcribed as a UGG codon, and the resulting mRNA is translated as the HDAg-L (Fig. 2B) (81, 82). The general ability of inosine to change the ORF in the viral RNA enables the virus to compress more genetic information into the same sequence. The possibility that other viruses utilize the same feature should be entertained, along with the potential immunogenic effects of this modification.

### 2′-*O*-methylations.

2′-*O*-methylations (Nm) (the addition of a methyl group to the 2′-OH of a ribose) of RNA have been a subject of interest since the 1960s, when they were discovered and observed by means of radioactive labeling of RNA (83, 84). Based on their position within the RNA molecule, 2′-*O*-methylations can be divided into two categories. The first category includes methylation of eukaryotic mRNA at the first and second nucleotide behind the 5′ cap (85). These structures, called Cap1 and Cap2 based on the position of the methylated nucleoside (86), are responsible for efficiency of processing, translation, overall stability, and susceptibility to degradation of mRNA (87, 88). Some viruses, such as coronaviruses, flaviviruses, orthomyxoviruses, and picornaviruses, rely on such a cap-dependent mechanism of translation. They can either use the host cell capping apparatus, snatch the caps from host mRNA (e.g., influenza), or code for their own capping machinery (89, 90). While the aforementioned viruses produce 5′ capped RNA, the lack of 2′-*O*-methylation may still alert the cell to their presence (91).

In fact, the absence of 2′-*O*-methylation on the first nucleotide of the 5′ cap (Cap0) is strongly immunogenic. The cytoplasmic pattern recognition receptor Mda5 is activated through binding to the Cap0 RNA (92). The activated Mda5 interacts with the mitochondrial antiviral signaling proteins (MAVS) through its N-terminal caspase activation and recruitment domains (CARDs). Working in a multiprotein complex, the MAVS recruit the inhibitor of nuclear factor kappa-B kinase subunit epsilon (IKKε) and the serine/threonine-protein kinase 1 (TBK1). This leads to the phosphorylation and transport of interferon regulatory factors 3 and 7 (IRF3 and IRF7) into the nucleus, where they activate the transcription of type I interferon genes IFN-α and IFN-β (Fig. 2) (93–97).

Another type of molecule capable of recognizing the Cap0 structure belongs to the IFIT family (interferon-induced proteins with tetratricopeptide repeats) (98). These molecules serve not only as detectors but also as effectors capable of inhibiting the viral life cycle (99). In particular, IFIT1 competes with the eukaryotic initiation factor 4E (eIF4E), which is part of the eukaryotic initiation factor 4F (eIF4F) that binds to the 5′ cap of mRNA (100). The eIF4E has a higher affinity for the Cap1 and Cap2 structures than IFIT1. On the other hand, IFIT1 has a higher affinity for the Cap0 structure. Binding to the viral RNA, IFIT1 leads to the abortion of viral translation (101). It also inhibits the formation of the 43S-mRNA complex and blocks the recruitment of eIF3 to the ternary complex, etc. (102).

The second type of 2′-*O*-methylation is present in internal RNA sites. These methylations are added to the viral RNA by hijacking the cellular methyltransferase FTSJ3. Cells in which the FTSJ3 methyltransferase is knocked down produce HIV-1 RNA with fewer methylations, and the virus induces higher expression of IFN-α and IFN-β (103).

It has been suggested and tested both *in vitro* and in mouse models that some RNA viruses may be attenuated by creating mutants lacking the Nm modification. This is done by creating recombinant viruses with a specific defect in the S-adenosylmethionine (SAM) binding site of the methyltransferase responsible for 2′-*O*-methylation. An infection with this recombinant virus elicits strong humoral and cellular immune reactions (104, 105). One of the model viruses used for this type of vaccine research was the severe acute respiratory syndrome coronavirus (SARS-CoV). Mutations introduced into nonstructural protein 16 (nsp16) created an attenuated virus by preventing it from creating the 2′-*O*-methylation (106). It also conclusively proved that viral Nm is an integral RNA modification necessary for a successful viral life cycle, and that viruses may utilize host methylating machinery to hide from the immune system. Given the recent outbreak of the disease COVID-19 associated with severe acute respiratory syndrome coronavirus 2 (SARS-CoV-2), it is definitely worthwhile to continue examining the modifications possessed by this virus and discover new ways of exploiting them for our benefit.

### Pseudouridine.

Pseudouridine (Ψ), often called the fifth nucleotide, is created by the isomerization of uridine. It is present in all RNA and in very high quantities in noncoding RNA (107). While the detection and location of pseudouridine in viral RNA is still in its infancy, and pseudouridine has yet to be detected in viral RNA, it has recently been reported that the enzyme pseudouridine synthase PUS7L is integral to the life cycle of HCV (108). An *in vitro*-prepared part of the polyU/UC RNA domain of HCV has been shown to act as a pathogen-associated molecular pattern that activates the pattern recognition receptor RIG-1 and leads to IFN-β production (109). The complete replacement of uridine with pseudouridine in this transcript drastically decreased IFN-β production, even though the RNA motif still had a high affinity for the RIG-1 molecule. Specifically, the RNA binds to RIG-1 but fails to trigger the conformational change associated with the activation of the molecule, thus disrupting the IFN-β immune response at an early stage (62). As it may be an essential part of the viral life cycle and the evasion of the host immune response, further research into the effect of pseudouridine in viral RNAs is warranted.

### Other RNA modifications.

Recently, a study based solely on liquid chromatography-mass spectrometry (LC-MS) analysis of viral RNA from ZIKV, dengue virus, HCV, poliovirus, and HIV-1 reported that the genomic RNA of these viruses contains, respectively, 32, 39, 42, 41, and 36 various chemical RNA modifications. Apart from numerous RNA modifications that were never before reported in mammalian systems, N^1^-methyladenosine (m^1^A) was also detected in all the tested viruses (110). Shortly thereafter, a study mapping m^1^A in RNA from the viral particle of HIV-1 showed that all the detected m^1^A comes from tRNA copacked in the viral particle, proving that HIV-1 genomic RNA does not contain m^1^A (22). This is in line with the finding that m^1^A is a typical tRNA modification and its presence in other types of RNA is somewhat rare (111).

Using ultraperformance liquid chromatography-tandem mass spectrometry (UPLC-MS/MS) analysis of the purified genomic viral RNA of two retroviruses, HIV-1 (65) and murine leukemia virus-MLV (66), it was recently shown that m^1^A, m^1^G, m^5^C, or m^7^G are present in viral RNA. These modifications, however, are present in tRNA^Lys^ and tRNA^Pro^, which serve as primers for the start of the reverse transcription of HIV-1 or MLV, respectively (13). It can be assumed that these tRNAs bind tightly to the genomic viral RNA. The sequencing libraries were prepared using the protocol for HIV-1 packageome analysis (112), which does not recover short RNAs (<50 nucleotides [nt]) or the highly structured tRNA. Even though the control sequencing analysis of the UPLC-MS/MS samples did not show any contamination from cellular RNA, the bound tRNAs could have been present and overlooked. It is important to note that, for example, 2-methylthio-N^6^-threonylcarbamoyladenosine (mS^2^t^6^A) causes a complete abortion of reverse transcription, and all the cDNA reads from tRNA^Lys^ have an approximate length of only 36 nucleotides and thus are not included in the library (22). Therefore, it would be useful to map m^1^A or m^1^G in retroviral genomic RNA with a profiling technique to confirm the presence of these modifications. It is important to note that modifications such as m^1^A and m^1^G would affect the function of the viral RNA because they disrupt traditional Watson-Crick base pairing, unlike m^6^A or m^5^C. For example, they may weaken complementary pairing of the molecule and change its coding capacity.

### Detection techniques.

Before a thorough study of the functions of a particular RNA modification, its existence and sequence position must be determined. Known viral RNA modifications and the methods of their detection are summarized in Table 1. Prior to the era of transcriptome sequencing (RNA-seq)-based techniques (113), mass spectrometry (MS) and radioactive labeling were commonly used to discover new RNA modifications. Even today, MS remains a very important tool capable of confirming the presence of almost all the chemical modifications (114). Nevertheless, it does not allow for the determination of the exact position of an RNA modification within the RNA sequence. The common procedure comprises the isolation of very pure target RNA material, followed by its digestion into the form of nucleosides or nucleotides. Analysis by means of MS usually requires a larger amount of starting material (isolated RNA) compared with RNA-seq-based methods. Although MS is a direct method and does not suffer from amplification bias created during library preparation, its main limitation lies in the purification of a particular RNA. Because rRNA represents about 85%, and tRNA about 12%, of cellular RNA (115), contamination of mRNA or viral RNA with these very abundant RNAs sometimes causes false positives when detecting RNA modifications.

**TABLE 1:** Summary of all detected RNA modifications in viral genomic RNA or viral mRNA with the techniques used for their detection and other potentially useful detection techniques

Modification	Type of virus by Baltimore classification and *family:* species	Type of viral RNA	Methods used for detection	Methods available for application
*N*^6^-methyladenosine m^6^A 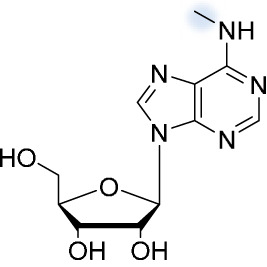	ss-RNA-RT, *Retroviridae*:			Analysis of unlabeled RNA: antibody based: (i) m^6^A-seq (20); (ii) MeRIP-seq (18); (iii) direct m6A seq (RT-KlenTaq DNA polymerase) (119); (iv) MAZTER-seq (120); (v) miCLIP (116); (vi) DART-seq (122) Metabolic labeling of cells: (i) metabolic propargyl labeling (130); (ii) PA-m^6^A-seq (antibody assisted) (129) Methods for the confirmation of m^6^A position: (i) SCARLET (121)
	Avian sarcoma viruses	Genomic, mRNA	^32^P-labeling (154–156), m^6^A-seq (52), PA-m^6^A-seq (49)	
	HIV-I	Genomic, mRNA	^32^P-labeling (154–156), m^6^A-seq (52), PA-m^6^A-seq (49)	
	Murine leukemia virus (66)	Genomic	UPLC-MS/MS, PA-m^6^A-seq	
	(−)ss-RNA, *Orthomyxoviridae*:			
	Influenza A (151)	Genomic, mRNA	PAR-CLIP with YTHDF (157)	
	Influenza A (151)	mRNA	PA-m^6^A-seq, radioactive labeling with [methyl-^3^H]methionine (158, 159)	
	ds-DNA, *Polyomaviridae*:			
	Simian virus 40 (152)	mRNA	PAR-CLIP with YTHDF, PA-m^6^A-seq	
	(+)ss-RNA, *Flaviviridae:*			
	Zika virus (58)	Genomic	UPLC-MS/MS, MeRIP-seq	
	HCV	Genomic	UPLC-MS/MS, MeRIP-seq	
	Dengue virus	Genomic	MeRIP-seq	
	Yellow fever virus	Genomic	MeRIP-seq	
	West Nile virus (57)	Genomic	MeRIP-seq	
	(+)ss-RNA, *Picornaviridae:*			
	Enterovirus 71 (153)	mRNA	MeRIP-seq	
5-methylcytidine m^5^C 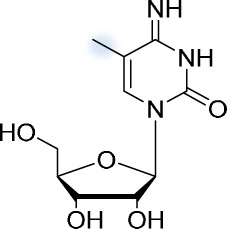	(+)ss-RNA, *Togaviridae*:			(i) Bisulfite sequencing (123–126); (ii) m^5^C-RIP (117); (iii) Aza-IP (131); (iv) miCLIP (132); (v) RBS-seq (128)
	Sindbis virus	mRNA	Radioactive labeling (60, 61)	
	ss-RNA-RT, *Retroviridae*:			
	HIV-I (65)	Genomic, mRNA	PA-m^5^C-seq	
	Murine leukemia virus (66)	Genomic	UPLC-MS, PA-m^5^C-seq	
	(+)ss-RNA, *Betacoronaviridae*:			
	SARS-CoV-2 (67)	Genomic, mRNA	Nanopore sequencing	
Inosine I 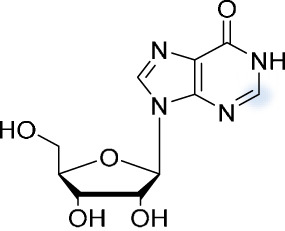	(−)ss-RNA, *Paramyxoviridae*:			(i) Sequencing comparison (133–135); (ii) ICE-seq (136)
	Measles virus	mRNA	Sequence comparison (164, 165)	
	ss-RNA-RT, *Retroviridae*:			
	HIV-I (74, 160)	mRNA	Sequence comparison	
	ds-DNA, *Herpesviridae*:			
	Human herpesvirus 8 (71)	mRNA	Restriction enzyme cleavage of cDNA	
	(+)ss-RNA, *Flaviviridae*:			
	Zika virus (58, 161, 162)	Genomic, mRNA	Sequence comparison	
	Subviral satellite, *Ïncertae sedis*:			
	HDV (70, 163)	Genomic, mRNA	Sequence comparison	
2′-*O*-methylnucleoside Nm 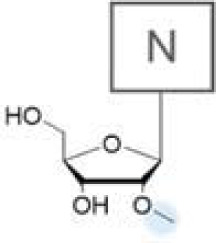	As part of cap:			(i) RiboMethSeq (139, 140); (ii) RibOxi-seq (141); (iii) Nm-seq (142, 143)
	All viruses use cap-dependent translation (98)			
	Internal location:			
	(+)ss-RNA, *Flaviviridae*:			
	Zika virus (U, C, G, and A) (58)	Genomic	UPLC/MS-MS	
	Dengue virus (166)	Genomic	UPLC/MS-MS, radioactive labeling	
	ss-RNA-RT, *Retroviridae*:			
	HIV-1 (103)	Genomic, mRNA	RiboMeth-Seq	

In contrast, RNA-seq methods allow for the determination of the exact position of RNA modifications within the entire transcriptome. The main disadvantage is the necessity of developing a specific capture/profile technique for every RNA modification. Once such a method is available, captured RNA is reverse transcribed into cDNA (which does not contain any modifications) and then amplified. The majority of methods rely on selective antibodies against m^6^A, including m^6^A-seq (20); MeRIP-seq (18); miCLIP (116); and m^5^C (m^5^C-RIP) (117). The main issue with antibody-based methods is the lack of specificity and effectivity of the antibodies used. Nonspecific binding of the antibodies often introduces significant bias into the results, so a careful approach is thus required (118). To overcome this problem, alternative techniques combined with next-generation sequencing have been developed for m^6^A profiling, such as employment of RT-Klentaq DNA polymerase (119) or, more recently, MAZTER-seq (120). Other techniques for the detection of m^6^A, such as SCARLET, require prior knowledge of the position of m^6^A (121). Another antibody-independent method is called DART-seq (deamination adjacent to RNA modification targets). This uses the m^6^A-binding domain YTH fused to the cytidine deaminase APOBEC1. The C nucleotides next to the m^6^A are deaminated into U, which is subsequently recognized using RNA-seq (122). The development of selective chemical techniques for m^6^A profiling, which would overcome all the disadvantages of the previous methods, is limited by the similar chemical structure and similar reactivity of m^6^A to canonical adenosine.

While there is an antibody-based approach for the detection of m^5^C, bisulfite sequencing is also frequently used. It relies on the selective chemical reaction of canonical cytidine and 5-methyl cytidine and is a functional alternative to the aforementioned antibody-based techniques (123–126). Relatively harsh reaction conditions, however, may destroy fragile RNA molecules, and other modifications (such as *N*^4^, 2′-*O*-dimethylcytidine) are sometimes mistaken for m^5^C. Moreover, the standard RNA bisulfite protocol has been shown to generate false-positive results when working with highly structured RNA (63). Nevertheless, the method has been used effectively to detect m^5^C in mouse embryonic and brain polyA RNA (127) and, recently, a modified bisulfite sequencing method called RBS-seq was introduced for simultaneous detection of Ψ, m^1^A, and m^5^C in a transcriptome-wide manner (128). To avoid the drawbacks of the aforesaid techniques, metabolic labeling methods were developed to detect both m^6^A (PA-m^6^A-seq [129] or metabolic propargyl labeling [130]) and m^5^C (Aza-IP [131] and miCLIP [132]). In general, the main disadvantage of metabolic labeling using propargyl, 5-azacytidine, or 4-thiouridine is that it introduces a major type of stress to the cell, such that the results do not represent the state of a healthy system.

Even though there are currently no antibody-based methods to detect inosine, a comparison of genomic sequences with the corresponding cDNA reveals its position within the RNA molecule. Inosine pairs with cytidine, and the cDNA thus contains a guanosine in its place (133). However, the method is prone to false positives, as it does not distinguish mapping errors, alignment errors, or single-nucleotide polymorphisms (134, 135). As an alternative, a chemical method called ICE-seq (RNA-seq based on the selective reaction of inosine with cyanoethyl) was developed in 2010 (136, 137).

There are no antibodies against 2′-*O*-methylation, and the modification is fairly unreactive. There are also two types of 2′-*O*-methylations distinguished by their position within the RNA molecule, where one is a part of the 5′ cap (85) and the other in internal RNA sites. The 2′-*O*-methylation within the cap structure can be detected mainly using UPLC-MS/MS (138). Identification of methylated internal sites is more complicated, and the developed techniques rely on a higher stability of the methylated position under basic conditions (RiboMethseq [139, 140]) or during oxidation (NaIO_4_) and β-elimination (RiboOxi-seq [141] and Nm-seq [142]). It was discovered, however, that in the case of less abundant RNA, Nm-seq is prone to mispriming and false positives (143).

In the future, all the problems caused by classical next-generation sequencing-based methods might be overcome with direct nanopore sequencing. This technique has the potential to detect RNA modifications directly without the need for reverse transcription and amplification. It has already been used in the sequencing of influenza virus RNA (144). In nanopore sequencing, the RNA moves through a pore and disrupts the electric current around it, causing so-called squiggles. Theoretically, every base and every modification disrupts the current differently and can thus be identified (145). Problems with alignment and current intensity changes, however, have prevented the creation of a successful detection algorithm. This issue can be circumvented by analyzing base-calling errors for some modifications, such as m^6^A, by comparing the target RNA with a nonmodified (or severely depleted) control RNA, or by employing artificial intelligence (AI) (146, 147).

**Conclusion and outlook.** While internal RNA modifications in mRNA or viral genomic RNA and mRNA do exist, they are not as diverse and abundant as many believed in 2012, when the field of epitranscriptomics was established. Nevertheless, the chemical modifications of viral RNA described above obviously play a role in the viral life cycle, in interactions with host innate immunity, and in the distinction between self and nonself RNA. Despite that several attempts have been made to exploit one of these modifications in order to create an attenuated vaccine in several viruses, to the best of our knowledge, no attempts to target the other modifications are currently being made. In comparison with internal modifications, new discoveries of modifications, such as the 5′ diphosphate termini in reoviruses, have shown that there is still much to be learned about the 5′ RNA moieties. The 5′ diphosphate RNA is recognized by RIG-I-like receptors in the cytoplasm and starts an antiviral cascade like the one described for the cap0 structure (107). Recently, a new mass spectrometry detection method called CapQuant has been used to identify several new types of RNA caps in purified dengue virions, including FAD (flavin adenine dinucleotide); UDP-Glc; UDP-GlcNAc; and m^7^Gpppm^6^A (108). We have recently described a new class of RNA caps in bacteria; dinucleoside polyphosphates were discovered using a combination of biochemical methods together with mass spectrometry (148). Given that some dsDNA viruses (e.g., poxviruses) encode their own NudiX enzymes that may process the 5′ dinucleoside polyphosphate RNA caps, the existence of such alternative caps in viral RNA cannot be ruled out (149, 150). The roles of most of these modifications and whether they are present in viral RNA remain to be determined. There is also a need for a careful and controlled approach when preparing RNA samples in order to generate reproducible and trustworthy data, or when applying a particular detection technique. Although new detection methods are constantly being developed based on ingenious new techniques, such as mutated enzymes that specifically interact with given modifications (120, 122), AI analysis of collected data, together with nanopore sequencing, seems the most promising. It is clear that viral RNA and the roles played by its modifications still hold a number of secrets. Thanks to the high abundance of viral RNA molecules in infected cells, they may well be a crucial model for understanding the role of similarly modified cellular RNA and further expanding our knowledge in the field of RNA modifications.
